# A reassessment of the Resistance to Framing scale

**DOI:** 10.3758/s13428-022-01876-7

**Published:** 2022-07-18

**Authors:** Sandra J. Geiger, Jáchym Vintr, Nikolay R. Rachev

**Affiliations:** 1 Department of Cognition, Emotion, and Methods, Faculty of Psychology, Environmental Psychology Unit, University of Vienna, Wächtergasse 1, 1010 Vienna, Austria; 2 Department of Psychology, Faculty of Arts, Charles University, Prague, Czech Republic; 3 Department of General, Experimental, Developmental, and Health Psychology, Sofia University St. Kliment Ohridski, Sofia, Bulgaria

**Keywords:** Resistance to Framing scale, Psychometrics, Validation, Confirmatory factor analysis, Item response theory

## Abstract

Risky-choice and attribute framing effects are well-known cognitive biases, where choices are influenced by the way information is presented. To assess susceptibility to these framing types, the Resistance to Framing scale is often used, although its performance has rarely been extensively tested. In an online survey among university students from Bulgaria (*N* = 245) and North America (*N* = 261), we planned to examine the scale’s psychometric properties, structural validity, and measurement invariance. However, some of these examinations were not possible because the scale displayed low and mostly non-significant inter-item correlations as well as low item-total correlations. Followingly, exploratory item response theory analyses indicated that the scale’s reliability was low, especially for high levels of resistance to framing. This suggests problems with the scale at a basic level of conceptualization, namely that the items may not represent the same content domain. Overall, the scale in its current version is of limited use, at least in university student samples, due to the identified problems. We discuss potential remedies to these problems, as well as provide open code and data (https://osf.io/j5n6f) which facilitates testing the scale in other samples (e.g., general population, different languages and countries) to obtain a comprehensive picture of its performance.

**Supplementary Information:**

The online version contains supplementary material available at 10.3758/s13428-022-01876-7.

We face many choices every day, each of which may be guided by subtle but powerful forces. One such force lies in the way information is presented. Consider the following thought experiment: Suppose you have invested $6,000 in stocks of a green energy company. As the economy is currently experiencing a downturn, you have two alternatives, one will save $2,000 of your investment, while the other one saves everything with a 33% chance and nothing with a 67% chance. Which of these strategies would you prefer?

Now, consider that you instead learned that one strategy will make you lose $4,000 of your investment, while the other one makes you lose nothing with a 33% chance and everything with a 67% chance (adapted from Bruine de Bruin et al., 2007). How would you choose based on this information?

Although the two descriptions are objectively equivalent, individuals typically choose the safe option (i.e., saving $2,000) when framed as gains rather than losses, and the risky option (i.e., losing nothing with a 33% chance and everything with a 67% chance) when framed as losses rather than gains. This well-established phenomenon is known as the *risky-choice framing effect*, whereby systematically different responses are elicited by the way the risk (gains vs. losses) is presented (Levin et al., 1998; Tversky & Kahneman, 1981). A related phenomenon occurs when characteristics of objects (e.g., treatment) are described in a positive (e.g., 50% success rate) or negative (e.g., 50% failure rate) way, which is known as the *attribute framing effect* (Levin et al., 1998; Levin & Gaeth, 1988).

## Resistance to risky-choice and attribute framing

The framing effect and its underlying mechanisms have been studied in various experimental contexts (e.g., Bloomfield, 2006; Fagley & Miller, 1997; Finn, 2008; Levin & Gaeth, 1988; Whitney et al., 2008) since it had first been demonstrated (Tversky & Kahneman, 1981). Thereby, competing claims about the framing effect and its mechanisms (e.g., Chick et al., 2016, Mandel & Kapler, 2018) are often based on a single task (e.g., variations of the Disease problem, Tversky & Kahneman, 1981; see Piñon & Gambara, 2005), limiting the reliability and validity of the findings. Different framing tasks in different domains (e.g., money or lives) are used to support these claims, which makes it difficult to compare the evidence for or against the competing viewpoints because the framing effect is sensitive to task content (Fagley & Miller, 1997). In addition, framing tasks are conceptualized for between-participants administration. This does not allow for calculating individual difference scores and thus investigating individual differences of framing effects (but see Mandel & Kapler, 2018).

The *Resistance to Framing* scale, a subscale of the Adult Decision-Making Competence Index (A-DMC; Bruine de Bruin et al., 2007), solves the above problems by using 14 positively and negatively framed tasks for both types of framing which vary by topic (e.g., money, human and animal lives, health). The scale thus potentially provides a unified (standard) measure of Resistance to Framing that is relatively content-independent. The within-participants administration of the scale allows for investigating individual differences by calculating individual scores. This within-participants administration is often used to identify cognitive predictors of Resistance to Framing, such as numeracy (Del Missier et al., 2012), cognitive ability (e.g., Raven’s standard progressive matrices; Bruine de Bruin et al., 2007), working memory (Hoffmann et al., 2020), cognitive and executive functions (e.g., cognitive flexibility and inhibitory control; Mäntylä et al., 2012, Piryaei et al., 2017), as well as cognitive reflection (Allred, 2018).

Although the scale is widely used, its performance has rarely been extensively tested. Previous research has exclusively tested the scale’s performance in terms of internal consistency and predictive validity in a US-American sample (Bruine de Bruin et al., 2007). Besides this research, several studies have validated the A-DMC as a whole and reported the internal consistency of the Resistance to Framing scale, which varied from Cronbach’s *ɑ* = .30 to .72 (Bavolar, 2013; Del Missier et al., 2012; Feng et al., 2013, as cited in Liang & Zou, 2018; Parker & Fischhoff, 2005). From this previous work, researchers have concluded that the Resistance to Framing Scale as part of the A-DMC is a valid and reliable instrument without extensively investigating its performance.

The factor structure underlying the Resistance to Framing scale remains subject to debate. When using the scale, researchers classically average over the absolute difference of the ratings in the positive and negative frame in each scenario (Bruine de Bruin et al., 2007). They thereby implicitly assume that the scale is unidimensional. This assumption is supported by evidence showing that risky-choice framing can be reduced to attribute framing, as systematically different choices in risky-choice framing scenarios result from framing effects of the riskless rather than the risky option (Kühberger & Gradl, 2013). Contrasting with this view, the original typology introduced by Levin et al. (1998) posits that risky-choice and attribute framing are distinct types based on what is framed (options with varying levels of risk vs. characteristics of an object), what is affected (risk preference vs. object evaluation), and how the effect is assessed (comparing choices of options vs. attractiveness of an object)—all of which are expected to influence the effect. Our reanalyses of previous data (Bavolar, 2013; Bruine de Bruin et al., 2007) before conducting this study supported two distinct types (Supplement 1).

The factor structure underlying the scale has not yet been investigated, although such an investigation is important because the standard practice of averaging over all absolute differences is only warranted as long as a one-factor model is empirically supported.

### The present study

The present study extensively tested the performance of the Resistance to Framing scale in samples from Bulgaria and North America. This additional assessment contributes to existing studies in three key ways: First, it goes beyond the internal consistency of the overall scale by using exploratory item response theory models. These models allow us to examine each item’s performance and the scale’s measurement precision across different levels of susceptibility to framing. Second, the present study examines the structural and cross-country validity of the scale using confirmatory factor analyses, as recommended in guidelines for evaluating measurement instruments (Chan, 2014). Last, the current results suggest that the scale performs poorly in university student samples and should not be used in its current version—a conclusion that challenges previous assumptions that the scale is valid and reliable.

## Method

To assess the performance of the Resistance to Framing scale, we conducted an online survey among Bulgarian and North American university students. We had preregistered our research questions, sampling plan, study design, and data analyses on the Open Science Framework (https://osf.io/uj5rz, September 11, 2020) before data collection. We assure that we reported how we determined our sample size, all data exclusions (if any), all manipulations, and all measures in the study (as suggested by Simmons et al., 2012). Deviations from the preregistration are reported in Supplement 2.

### Sampling plan

Our preregistered target sample size was 250 complete responses per site after excluding responses categorized as duplicate, inattentive, or too fast (see Data exclusions). The data were collected as part of a larger research project to evaluate the dual-process account in Bulgaria and North America (Rachev et al., 2022). The sample size has been determined based on the fit and misfit of the confirmatory factor analysis (CFA) models and expected effect sizes used within this larger project. A priori power analyses for the present study with the R package *semTools* (Version 0.5-3; Jorgensen et al., 2020) indicated that 250 participants per site allowed us to detect fit and misfit of a one- (Resistance to Framing) and two-factor (risky-choice and attribute framing) model with more than 95% power (Supplement 1).^1^

### Participants

Participants were required to be at least 18 years old, citizens and residents of either Bulgaria or North America, native Bulgarian or English speakers, and university students with a non-psychological major. Characteristics of both samples are in Table 1.

**Table 1:** Sample characteristics (Bulgaria and North America)

	Bulgaria	North America
Full sample size	375	392
Final sample size ^a^	245	261
Age (in years)	M = 23.2, SD = 6.2, Range = 18–52	M = 21.9, SD = 4.2, Range = 18–49
Proportion female (in %)	78.4	42.9
Residence	100% Bulgaria	75.9% United States 24.1% Canada
Highest completed education	High school: 200 3-year bachelor’s: 13 4-year bachelor’s: 19 Master’s: 13	Below high school: 1 High school: 61 College: 123 2-year degree: 28 4-year degree: 41 Professional degree: 6 Doctorate: 1
Most common study programs	Philology: 49 Economics: 27 Computer science: 26	Computer science: 40 Engineering: 33 Biology: 21
Study Year	1^st^: 80 2^nd^: 64 3^rd^: 42 4^th^: 33 5^th^+: 26	1^st^: 44 2^nd^: 59 3^rd^: 53 4^th^: 54 5^th^+: 51

^a^After excluding data categorized as duplicate, inattentive, too fast, and multivariate outliers.

### Materials

The Resistance to Framing scale (Bruine de Bruin et al., 2007) consists of seven risky-choice and seven attribute framing items, with one positively and one negatively framed version per item. An example item of risky-choice framing is deciding between two investment strategies, as in the introduction of this paper. Participants indicate the strength of their preference for either strategy on a six-point scale with 1 *Definitely would choose A* and 6 *Definitely would choose B*. Resistance to Framing is then assessed by the absolute difference between the ratings for the positive and negative frame of every item (Bruine de Bruin et al., 2007), whereby lower scores represent lower susceptibility to framing.^2^ Compared to the original scale, we slightly modified the phrasing of the instruction and two scenarios to make their meaning clearer.

The Bulgarian version of the Resistance to Framing scale was created from the original English version by five (risky-choice framing) and four (attribute framing) independent translators using forward and backward translation. In particular, three (risky-choice framing) and two (attribute framing) experienced translators translated the original items to Bulgarian and discussed discrepancies to arrive at a joint forward translation. After establishing the same procedure for the back translation with two additional translators, all translators and an additional adjudicator (the third author) compared the original version to the back translation and resolved discrepancies. Pilot-testing before data collection did not reveal any concerns about the items or response format.

### Procedure

Participants in both sites were recruited from November 2020 to January 2021 via social media, emails, and universities. They were informed about the confidentiality and anonymity of their responses and provided written informed consent before starting the online survey. All individuals participated voluntarily and could determine their participation at any given moment. The study was approved by both local research ethics committees.

Participants completed the 30-min Qualtrics survey in their native language. They first created a withdrawal code and provided basic demographic information (i.e., age, gender, education, field and year of study, country of residence, citizenship, and native languages). Participants were then presented with either the positive or negative version of the risky-choice followed by the attribute framing block before being presented with the remaining versions. The framing problems within a block were presented in randomized order and forced-response format. Between the positive and the negative version, participants took approximately 10 min to complete the *Bullshit Receptivity scale* (Pennycook et al., 2015), the *Actively Open-Minded Thinking scale* (Baron & High II, 2019), and an attention check item (“Please select the option ‘neutral’ and proceed to the following question.”), all of which were part of the larger project. The survey ended with a debriefing and redirection to a separate survey where participants’ email addresses were recorded to enter a random draw of 165 (160 × $3 and 5 × $20) gift cards for Bulgarian participants and 90 (85 × $5 and 5 × $30) gift cards for North American participants.

### Statistical analysis

All analyses were conducted using R (Version 4.0.2; R Core Team, 2020) with the statistical significance level set to *α* = .05.

#### Data exclusions

Participants were included in the analysis if they answered all items, provided informed consent, and completed the survey only once (i.e., unique combination of IP address, withdrawal code, and demographics including age, gender, major, and year of studies; Koo & Skinner, 2005). Participants were further included if they passed the attention check and did not complete the survey faster than 10 min.^3^ In the Bulgarian and North American sample, 30 and 29 multivariate outliers, respectively, were excluded based on the generalized Cook’s distance using the R package *influence.SEM* (Version 2.2; Altoé & Pastore, 2018). Analyses including outliers are in Supplement 3.

#### Main analysis

We first inspected the descriptive statistics of each item, including mean, standard deviation, range, skewness, kurtosis, and inter-item as well as item-total correlations. To evaluate the factor structure, we fit a one-factor and two-factor CFA model in each site using the R package *lavaan* (Version 0.6-6; Rosseel, 2012).^4^ The fit of each model was assessed using several fit indices (Table 5) and their preregistered standard cut-off values.^5^ However, we refrained from comparing the models and from testing measurement invariance between sites, due to the low inter-item correlations (see Results). We used McDonald’s ω to assess the scale’s reliability as well as exploratory, non-preregistered item response theory (IRT) to get further insights into the scale’s measurement precision and the performance of each item. The IRT models were fit with the *mirt* package in R (Version 1.33.2, Chalmers, 2012) and full-information maximum likelihood estimation to account for the relatively small sample sizes (Forero & Maydeu-Olivares, 2009). We fit a *graded response* (Samejima, 1969) and a *generalized partial credit model* (Muraki, 1992), two commonly used polytomous models that apply to the data at hand. These models were compared based on the AIC and BIC as well as their sample-size adjusted counterparts (AICc and SABIC), and their fit was assessed based on multiple indices (Table 6).

## Results

### Descriptive statistics

Table 2 shows the descriptive statistics for both samples. Susceptibility to framing was generally low, especially for the attribute framing items. This was also supported by the skewness which indicated that all items were highly positively skewed (skewness > 1). Most inter-item correlations were small and non-significant (Tables 3 and 4). Item-total correlations (i.e., correlations between each item and the total score of all items excluding this item) ranged from .09 to .23 in Bulgaria and .04 to .32 in North America, which is below the recommended .30 (Boateng et al., 2018).

**Table 2:** Descriptive statistics, reliability, and factor loadings of the Resistance to Framing Scale (Bulgaria and North America)

	Bulgaria						North America					
	M (SD)	S	K	Factor Loadings			M (SD)	S	K	Factor Loadings		
				One-factor model	Two-factor model					One-factor model	Two-factor model	
				RtF	RCF	AF				RtF	RCF	AF
(1) Pesticide	1.38 (1.43)	1.01	0.17	**0.26**	**0.27**		1.13 (1.28)	1.21	0.82	**0.25**	**0.25**	
(2) Income tax	0.81 (1.06)	1.30	1.06	0.17	**0.19**		0.90 (1.07)	1.41	1.92	**0.40**	**0.38**	
(3) School drop-outs	1.02 (1.24)	1.44	1.76	**0.34**	**0.35**		0.85 (1.14)	1.45	1.65	**0.31**	**0.34**	
(4) Asian disease	1.02 (1.14)	1.23	1.19	**0.32**	**0.34**		1.17 (1.32)	1.11	0.42	**0.72**	**0.77**	
(5) Cancer treatment	0.93 (1.26)	1.41	1.18	**0.28**	**0.27**		0.90 (1.32)	1.75	2.47	0.20	0.18	
(6) Stocks	0.99 (1.20)	1.51	2.09	**0.25**	**0.27**		0.93 (1.13)	1.23	0.90	**0.57**	**0.57**	
(7) Soldiers	1.02 (1.19)	1.24	1.01	**0.38**	**0.41**		1.09 (1.20)	1.34	1.64	**0.45**	**0.45**	
(8) Condom	0.42 (0.73)	2.15	5.36	**0.25**		**0.26**	0.40 (0.65)	1.77	3.43	0.04		0.10
(9) Ground beef	0.67 (0.88)	1.46	2.02	**0.22**		**0.22**	0.49 (0.74)	1.58	2.57	0.06		**0.26**
(10) Cheating	0.58 (0.92)	1.73	2.65	0.10		0.09	0.48 (0.73)	1.61	2.41	**0.11**		**0.21**
(11) Project budget	0.52 (0.74)	1.44	1.72	**0.20**		**0.20**	0.50 (0.69)	1.24	0.99	**0.14**		0.11
(12) Exam	0.41 (0.66)	1.60	2.16	**0.24**		**0.24**	0.30 (0.51)	1.39	0.94	0.06		**0.14**
(13) Illegal parking	0.53 (0.83)	1.94	4.24	**0.26**		**0.27**	0.52 (0.72)	1.36	1.63	**0.16**		**0.34**
(14) Cancer family	0.42 (0.63)	1.22	0.33	**0.17**		**0.17**	0.36 (0.58)	1.70	3.65	0.01		0.05

*RtF* Resistance to Framing, *RCF* risky-choice framing, *AF* attribute framing, *M* mean absolute difference between the positive and negative frame, *SD* standard deviation, *S* skewness, *K* kurtosis. Bold font indicates that this item loaded significantly onto the corresponding factor.

**Table 3:** Inter-item and item-total correlations (Bulgaria)

Item	1	2	3	4	5	6	7	8	9	10	11	12	13	ITC
Risky-Choice														
1. Pesticide														.14
2. Income tax	.00													.11
3. School dropouts	.06	.02												.19
4. Asian disease	-.01	.09	.10											.19
5. Cancer treatment	.04	.02	.00	.03										.11
6. Stocks	.08	.03	.15*	.14*	-.02									.17
7. Soldiers	.11	.13*	.05	.11	.12	.02								.23
Attribute														
8. Condom	-.02	.02	.10	.03	.08	-.06	.19**							.17
9. Ground beef	.14*	.02	.11	.13*	.07	.05	.00	.05						.18
10. Cheating	.07	.03	.03	.01	-.05	.13*	.13*	.05	-.08					.09
11. Project budget	.11	-.02	.07	.11	.06	.03	.02	.04	.08	.03				.18
12. Exams	.01	.01	.08	.13*	.12	.06	.13*	.23**	.11	-.05	.06			.21
13. Illegal parking	.14*	.02	.04	.03	.08	.10	.07	.13*	.12	.08	.15*	.08		.22
14. Cancer family	-.07	.18**	.12	.06	.07	.08	.06	.12	-.01	.02	.14*	.05	.08	.18

*Note.* ITC item-total correlation. * indicates *p* < .05. ** indicates *p* < .01.

**Table 4:** Inter-item and item-total correlations (North America)

Item	1	2	3	4	5	6	7	8	9	10	11	12	13	ITC
Risky-Choice														
1. Pesticide														.15
2. Income tax	.07													.24
3. School drop-outs	.02	.15*												.17
4. Asian disease	.14*	.22**	.20**											.32
5. Cancer treatment	– .02	– .03	.07	.06										.13
6. Stocks	.07	.19**	.09	.31**	.06									.30
7. Soldiers	.08	.08	.15*	.20**	.13*	.19**								.28
Attribute														
8. Condom	.09	.06	.06	.03	.04	– .05	– .04							.09
9. Ground beef	– .00	.04	– .04	– .10	.08	.04	.13*	.01						.11
10. Cheating	.03	.15*	– .03	– .00	– .02	.08	.09	.01	.14*					.12
11. Project budget	.06	.10	.02	.12	.04	.04	.11	.06	.01	.06				.15
12. Exams	.12	.10	– .05	– .01	– .00	.09	– .02	.10	.12	.13*	– .01			.12
13. Illegal parking	– .02	.04	– .03	.08	.21**	.17**	.04	.07	.19**	.10	.08	.11		.22
14. Cancer family	.12	– .02	– .03	.03	.04	– .03	.02	.06	.03	– .07	– .05	– .01	.11	.04

*ITC* item-total correlation.* indicates *p* < .05. ** indicates *p* < .01.

**Fig. 1:**
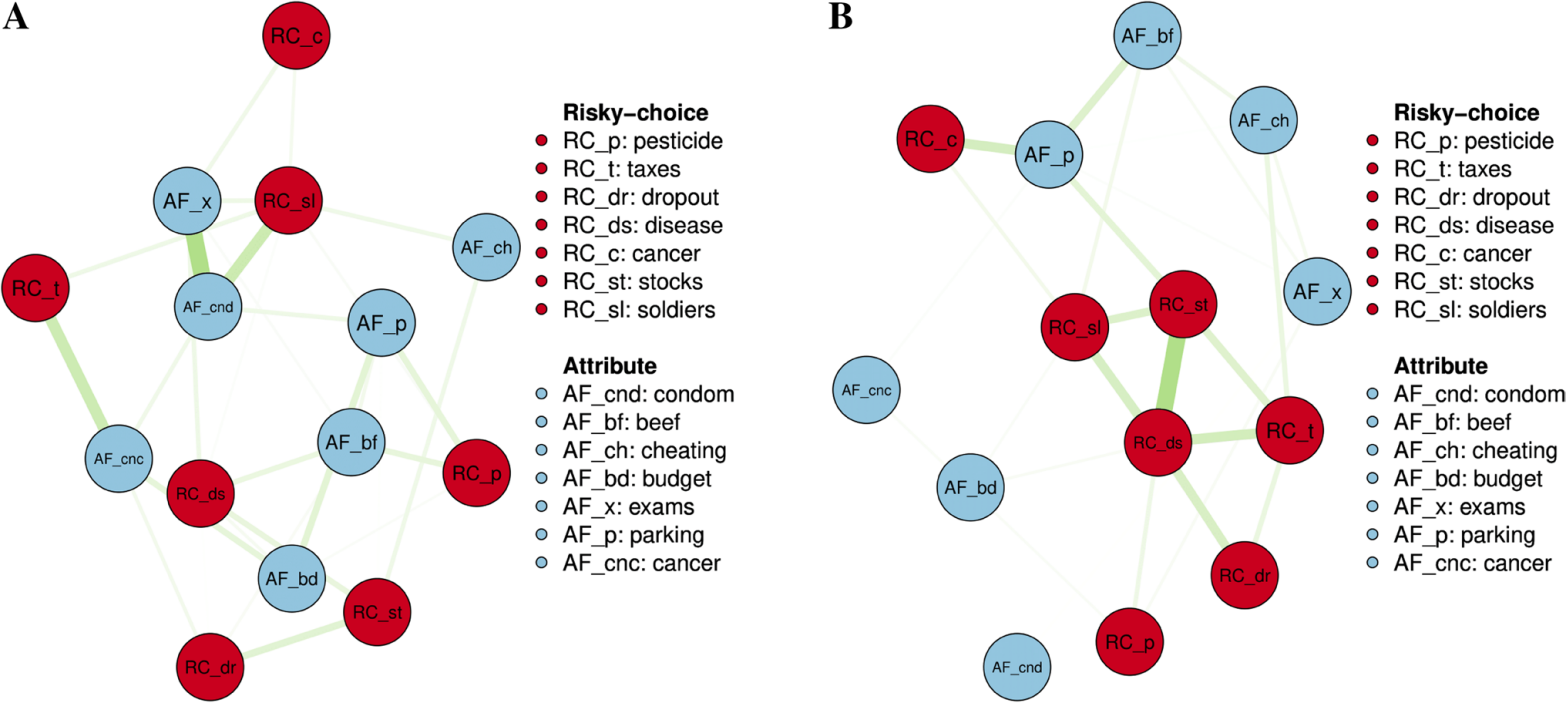
Network plots of the bivariate correlations between the items of the Resistance to Framing scale for **A** Bulgaria and **B **North America. Only bivariate correlations larger than *r* = .10 are displayed. Red edges indicate a negative correlation between two items, whereas green edges indicate a positive correlation

The network plots in Fig. 1 additionally visualize that few inter-item correlations are larger than *r* = .10. Moreover, we would expect either that all items of the scale are substantially positively correlated (which would point toward a one-factor model) or that the items form two clusters, risky-choice and attribute framing (which would point toward a two-factor model). However, neither pattern seems to be supported.

### Structural validity

Inspecting the bivariate correlations already revealed that there may be problems with the structure of the scale. We nevertheless proceeded with the preregistered plan and fit one- and two-factor CFA models. The models’ structure was determined based on theory (see introduction) and the classification of the items into risky-choice and attribute framing proposed in the original article (Bruine de Bruin et al., 2007).

The hypothesized one- and two-factor model fit the Bulgarian data acceptably, based on the robust χ^2^-test, the robust RMSEA, and robust incremental fit indices (Table 5). However, in the one-factor model, two of 14 items (i.e., risky-choice: income tax and attribute framing: cheating) did not load significantly onto the factor. This problem persisted in the two-factor model where the attribute framing item cheating still did not load significantly onto its corresponding factor. More worryingly, all standardized factor loadings (Table 2) were low and below the recommended cut-off of .45 (Tabachnick et al., 2007).

**Table 5:** Model fit of the one- and two-factor model in the Bulgarian and North American sample

	χ^2^(*df*)	***p***	RMSEA	CFI	TLI/NNFI	IFI
Bulgaria						
M1: 1-Factor Model	66.11 (77)	.807	.00 [.00, .03]	1.00	1.32	1.20
M2: 2-Factor Model	65.73 (76)	.794	.00 [.00, .03]	1.00	1.31	1.18
North America						
M3: 1-Factor Model	92.65 (77)	.108	.03 [.00, .05]	0.83	0.80	0.86
M4: 2-Factor Model	78.69 (76)	.394	.01 [.00, .04]	0.97	0.97	0.98

RMSEA Root mean square error of approximation, *CFI* Comparative Fit Index, *TLI* Tucker–Lewis Index, *NNFI* Non Normed-Fit Index, *IFI* Incremental Fit Index. The interval around the RMSEA represents the 90% confidence interval.

The hypothesized one-factor model did not fit the North American data acceptably (Table 5), and five of the 14 items (i.e., risky choice: cancer, attribute framing: condom, beef, exams, and cancer) did not load significantly onto the factor. While the two-factor model fit well (Table 5), four of the 14 items (i.e., risky-choice framing: cancer, attribute framing: condom, budget, and cancer) still did not load significantly onto their corresponding factor. Once again, the factor loadings were small, and only three out of 14 met the recommended criterion. As such small factor loadings indicate problems at a basic level of conceptualization and may explain the very good model fit (reliability paradox; McNeish & Wolf, 2021), we refrained from comparing the models in each site and from testing measurement invariance between sites.

#### Reliability

The Bulgarian one-factor model indicated poor reliability (McDonald’s ω = .46), similar to the two-factor model with risky-choice (McDonald’s ω = .31) and attribute (McDonald’s ω = .35) framing. This corresponds to the scale’s reliability in the North American sample (McDonald’s ω = .51) and the two separate scales, risky choice (McDonald’s ω = .50) and attribute framing (McDonald’s ω = .34).

### Non-preregistered IRT models

#### Assumption checks

To get more detailed insights into the performance of each item, we fit an exploratory polytomous graded response and generalized partial credit model. Polytomous IRT models were used, as the Resistance to Framing scale may not be unidimensional (see CFA results). In the Bulgarian sample, both assumptions for fitting IRT models were met: local independence (i.e., items are uncorrelated when conditioning on the latent traits), as evidenced by relatively low discrimination parameters and residual covariances, and monotonicity (i.e., increased probability of endorsing an item constantly goes hand in hand with an increase in the latent trait; Nguyen et al., 2014). In the North American sample, the assumption of monotonicity was violated, with serious violations for the risky-choice item Dropout (*crit* = 88), unclear violations for five items (40 ≤ *crit* ≤ 80; attribute framing: condom, beef, cheating, exams, and cancer), and no violations for the other eight items (Molenaar & Sijtsma, 2000, as cited in Crișan et al., 2019). The graphical analyses corroborated these statistical results. As monotonicity was likely violated, no IRT models were fit to the North American data.

#### Model comparison

Comparing both models showed that the graded response model fit the Bulgarian data better than the generalized partial credit model. As displayed in Table 6, AIC and BIC as well as their sample-size adjusted analogs were lower for the graded response model. The other fit statistics indicated that both models fit the data well, as shown by a non-significant M2* value, a low RMSEA and SRMSR, as well as a high TLI (except for the graded response model) and CFI. The S−χ^2^ statistic indicated no misfit for any of the items of the graded response model but misfit for the risky-choice dropout item (S−χ^2^(40) = 59.741, *p* = .023) when using the generalized partial credit model. We thus selected the graded response model to examine the reliability, test information, and standard errors of the scale.

**Table 6:** Fit of the graded response and generalized partial credit model (Bulgaria)

Model	AIC	AICc	SABIC	BIC	M2*(*df*)	*p*	90% CI RMSEA	SRMSR	TLI	CFI
Graded response	7908	7973	7932	8167	32.61 (32)	.437	.01 [.00, .05]	.06	0.87	0.91
Generalized partial credit	7936	8002	7961	8195	24.01 (32)	.844	.00 [.00, .03]	.06	2.66	1.00

*AIC* Akaike Information Criterion, *AICc* sample-size adjusted AIC, *BIC* Bayesian Information Criterion, *SABIC* sample-size adjusted BIC, *RMSEA* Root mean square error of approximation, *SRMSR* Standardized root mean square residual, *TLI* Tucker–Lewis Index, *CFI* Comparative Fit Index. The interval around the RMSEA represents the 90% confidence interval.

**Fig. 2:**
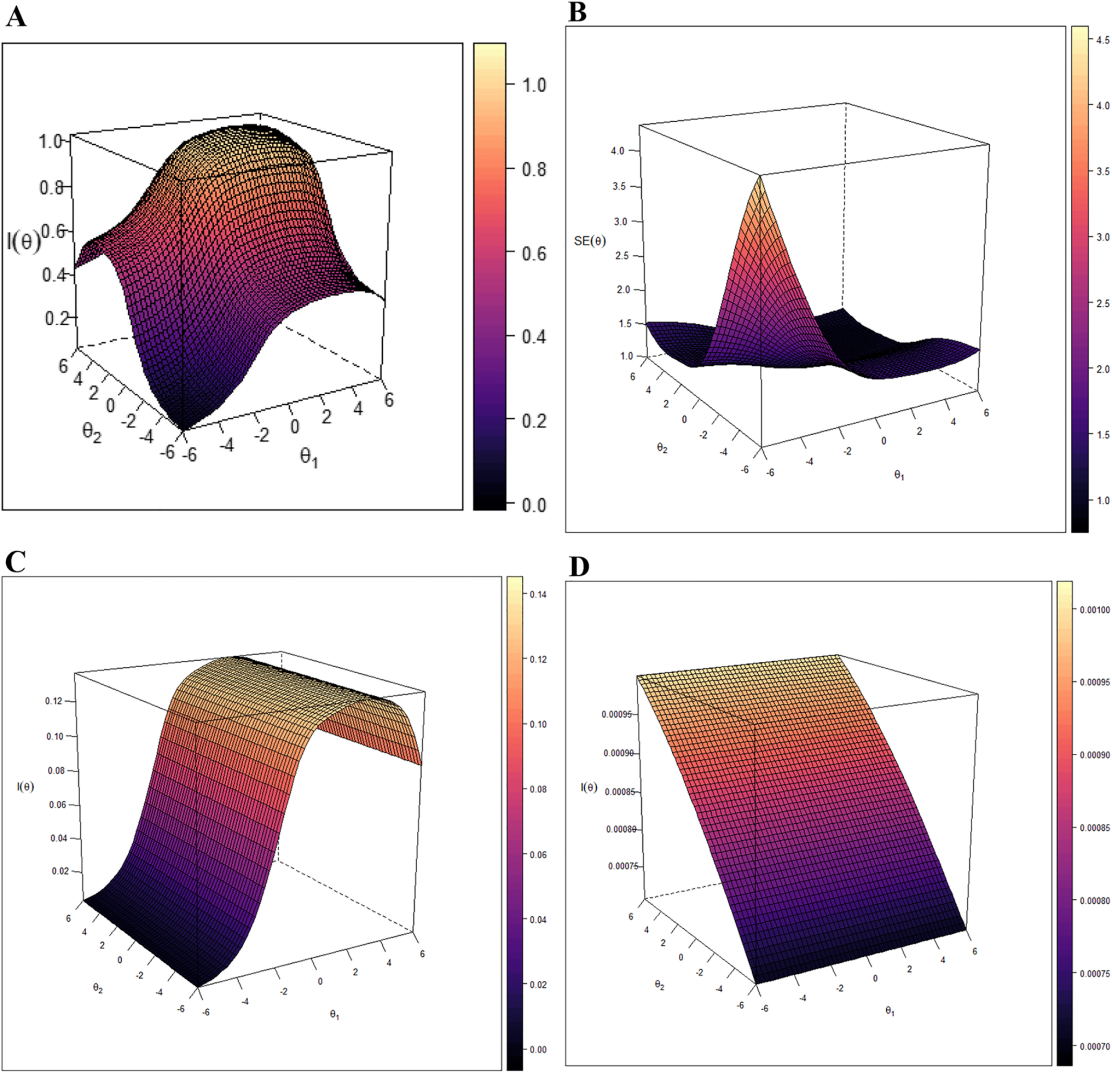
Test information function curve and test standard error for risky-choice (θ_1_) and attribute (θ_2_) framing (Bulgaria). Panel **A** displays the test information function curve for all items combined. Panel **B** displays the corresponding test standard error. Panel **C** and **D** display the test information function curve for the risky-choice framing item Disease and the attribute framing item cheating

In line with the reliability based on classical test theory, the IRT reliability was poor for risky-choice (0.52) and attribute framing (0.52). The test information in Fig. 2 shows how accurately the two subscales measure the latent traits, susceptibility to risky-choice (θ_1_) and attribute (θ_2_) framing. More test information thereby means higher measurement precision and lower standard errors of the underlying trait estimate (Nguyen et al., 2014). The test information was high for high levels of susceptibility to risky-choice and attribute framing, whereas it was very low for low levels (panel A). This means that high levels of susceptibility to framing can be measured with low standard errors and thus high precision, whereas low levels cannot be measured accurately (panel B). The test information of single items showed similar patterns: high test information and low standard errors for high levels of susceptibility to risky-choice and attribute framing, while for some items (e.g., risky-choice framing: disease), the test information decreased, and the standard error increased for extremely high levels of the underlying trait. The individual analysis of the test information function curves showed that among all items, the test information was highest for the two risky-choice framing items Disease (panel C) and Soldiers as well as the attribute framing item Parking. The test information and discriminating power (as indicated by a flat slope) were the lowest for the attribute framing item Cheating (panel D).

## Discussion

This study tested the performance of the Resistance to Framing scale. In an online survey among Bulgarian (*N* = 245) and North American (*N* = 261) university students, we planned to examine the scale’s psychometric properties, structural validity, and measurement invariance between sites. However, some of these examinations were not possible because the scale displayed low and mostly non-significant inter-item correlations as well as low item-total correlations. This suggests problems with the scale at a basic level of conceptualization, namely that the items may not represent the same content domain (Piedmont, 2014).

Confirmatory factor analyses suggested that the one- (one Resistance to Framing factor) and two-factor (resistance to risky-choice framing and resistance to attribute framing) model fit the data. However, this good fit may be caused by the low inter-item correlations, low factor loadings, and low reliability (reliability paradox; McNeish & Wolf, 2021). We, therefore, cannot make any conclusions about the factor structure, except that based on the inter-item correlations neither of the structures seemed to be supported.

The reliability was low in both sites, meaning that the individual items are only weakly related to each other and that participants’ observed scores do not reflect their true susceptibility to framing. The scale’s reliability in the present study was especially low compared to previous estimates. This could have resulted from the homogeneous nature of our student samples which displayed relatively low susceptibility to framing, thereby restricting variability and reducing reliability (Hedge et al., 2018). This could have also resulted from the short time interval between the two framing blocks which may have made it easy for participants to recognize that the task pairs are objectively equivalent and respond consistently across frames (but see Aczel et al., 2018). Exploratory item response theory analyses in the Bulgarian sample suggested that the scale can measure high levels of susceptibility to framing relatively accurately, while it is inaccurate for low levels. This means that the scale should be used with caution especially in samples where low susceptibility to framing is expected, for example presumably university students.

In addition, low reliability may result in biased correlation estimates between Resistance to Framing and other measures (Nimon et al., 2012). To correct for measurement error, we recommend computing disattenuated correlations or using latent variable modeling when using the scale. However, we would like to emphasize that this solution does not capture the full effect of measurement error on results. The most optimal way would be to improve the scale’s reliability, particularly since poor reliability has been a pervading problem of the Resistance to Framing scale and also of similar measures (e.g., Stanovich et al., 2016). Recently, a new risky-choice framing scale has become available (Berthet, 2021). Berthet was able to improve the scale’s reliability (Cronbach’s α 0.15 in Study 1 vs. 0.74 in Study 2) by increasing the number of items from four to eight and by slightly tweaking the cover stories in each pair of framing tasks (e.g., an accident caused by an autonomous vehicle vs. a train), while keeping the numbers and overall topic identical. The same approach may be applied to improve the reliability of both risky-choice and attribute framing of the Resistance to Framing scales. A positive result may not only represent good news for framing researchers but also point out that using identical tasks is suboptimal for within-participants designs.

An additional problem concerns the scoring of the scale. The original scoring system, also adopted in similar framing measures (Stanovich et al., 2016), is to average over the absolute differences in ratings of paired framing tasks. However, the framing effect is directional (see also Berthet, 2021), that is, higher risk-seeking is expected in the loss versus the gain frame, and higher approval is expected when an object is described in positive versus negative terms.^6^ Based on theory, it is reasonable to use directional rather than absolute differences. Directional scoring is problematic, however, because inconsistencies in opposite directions cancel each other out, which underestimates susceptibility to framing in individuals who display “negative” framing effects on certain problems. Not surprisingly, Berthet (2021) reported that absolute scoring increased the internal consistency of his risky-choice framing scale relative to directional scoring. This paradox of better theoretical justification leading to less optimal measurement should be kept in mind when using the scale. As LeBoeuf and Shafir (2003) noted, within-participants administration of the same framing tasks measures the ability to stay consistent across frames, not the ability to resist the initial influence of the frame. The choice of the scoring system may, therefore, depend on what should be measured, and which question should be answered.

### Limitations

This study has several limitations. Firstly, we found no clear answers to the questions of whether the scale’s structure and measurement invariance, due to low inter-item correlations. Secondly, we did not assess the convergent and discriminant validity of the scale, which would have allowed for a more comprehensive assessment, especially of the psychometric properties. Lastly, generalizability may be limited due to homogenous samples including highly educated, young, and in Bulgaria mainly female individuals. Generalizability may be further limited due to the short time frame between the two administrations and the relatively high—although for online surveys common—dropout rate (Galesic, 2006).

### Summary

In sum, this study points to three potential problems with the Resistance to Framing scale. First, low inter-item and item-total correlations suggest that the items of the scale may not represent the same content domain. Second, due to these low correlations, it remains unclear whether the scale’s factor structure is one- or two-dimensional. Last, the low reliability of the scale may prevent accurate measurement of susceptibility to framing, especially among highly resistant individuals. By providing open code and data (https://osf.io/j5n6f), we support researchers in testing the scale with other samples (e.g., general population, different languages and countries) to obtain a comprehensive picture of its performance.

## Supplementary Information

ESM 1 (DOCX 404 kb)

## Data Availability

This work was preregistered on the Open Science Framework (OSF; https://osf.io/uj5rz). The datasets generated during and/or analyzed during the current study are available in the OSF (https://osf.io/j5n6f).

## Footnotes

1 These analyses were based on a root mean square error of approximation (RMSEA) cutoff value of ≤ .08 for acceptable fit and a true RMSEA of .04 (Bavolar, 2013; Bruine de Bruin et al., 2007) for the one-factor model and .01 (Bavolar, 2013) for the two-factor model.

2 In most studies, lower scores represent lower Resistance to Framing rather than susceptibility to framing by subtracting the mean absolute difference from the potential maximum (5).

3 We deviated from our preregistered criterion of 3.5 median absolute deviations of the overall time (Leys et al., 2019) because many participants completed the survey faster than 10 min (*n* = 51 of 341 in North America and *n* = 7 of 282 in Bulgaria). Not changing the outlier criterion would have led to a low median and including many responses with an unreasonably short completion time.

4 We used robust maximum-likelihood estimation, as multivariate normality was violated according to the Henze-Zirkler test (*MVN*, Version 5.8, Korkmaz et al., 2014; Bulgaria: *HZ* = 1.43, *p* < .001, North America: *HZ* = 1.58, *p* < .001).

5 We planned to evaluate model fit based on dynamic fit indices (McNeish & Wolf, 2021), as models with a lowly compared to a highly reliable scale attain better model fit when using the standard fixed cutoff values (Hu & Bentler, 1999), the so-called reliability paradox (Hancock & Mueller, 2011). Dynamic fit indices were, however, not available for these models (https://www.dynamicfit.app/cfa/); so, we used the standard cut-off values which were preregistered as a backup plan.

6 This point was also raised by Jonathan Baron in a personal communication.

## Code Availability

The code used to prepare and analyze the data can be found on the OSF (https://osf.io/j5n6f). We used R (Version 4.0.2; R Core Team, 2020) and the R packages base (R Core Team, 2020), dplyr (Wickham, François, et al., 2020), ggplot2 (Wickham, 2016), grateful (Rodriguez-Sanchez, 2018), influence.SEM (Altoé & Pastore, 2018), lavaan (Rosseel, 2012), mirt (Chalmers, 2012), mokken (Van der Ark, 2007), MVN (Korkmaz et al., 2014), parameters (Lüdecke et al., 2020), pastecs (Grosjean & Ibanez, 2018), psych (Revelle, 2019), purrr (Henry & Wickham, 2020), readxl (Wickham & Bryan, 2019), Routliers (Delacre & Klein, 2019), sem (Fox et al., 2020), semPlot (Epskamp, 2019), semTools (Jorgensen et al., 2020), stringi (Gagolewski, 2020), tidyr (Wickham et al., 2020), tidyverse (Wickham  et al. 2019), and viridis (Garnier, 2018).
