# Supplementary material for: A reassessment of the Resistance to Framing scale
Source: Behav Res Methods. 2022 Jul 18;55(5):2320–32. doi: 10.3758/s13428-022-01876-7 (PMC10439025; doi:10.3758/s13428-022-01876-7)
Supplement: Supplementary file 1 — (DOCX 404 kb) [file 13428_2022_1876_MOESM1_ESM.docx]

# Supplement 1: Reanalysis of previous Data and Power Analyses

**Data Slovakia**

*# read the dataset*
path <- "data_slovakia.sav"
data <- foreign**::read.spss**(path, use.value.labels = T, to.data.frame = T)

## re-encoding from CP1250

*# only framing items*
d <- data **%>%** dplyr**::select**(**starts_with**(**c**("RC", "A"))) **%>%** dplyr**::select**(**-c**(ADR, age))

*# calculate difference scores*
dd <- d **%>%**
 **mutate**(RTF_RC1 = **abs**(RC11**-**RC25), RTF_RC2 = **abs**(RC12**-**RC24), RTF_RC3 = **abs**(RC13**-**RC27), RTF_RC4 = **abs**(RC14**-**RC22), RTF_RC5 = **abs**(RC15**-**RC26), RTF_RC6 = **abs**(RC16**-**RC23), RTF_RC7 = **abs**(RC17**-**RC21), RTF_A1 = **abs**(A11**-**A26), RTF_A2 = **abs**(A12**-**A25), RTF_A3 = **abs**(A13**-**A23), RTF_A4 = **abs**(A14**-**A21), RTF_A5 = **abs**(A15**-**A27), RTF_A6 = **abs**(A16**-**A22), RTF_A7 = **abs**(A17**-**A24)) **%>%**
 dplyr**::select**(**starts_with**("RTF"))

*# multivariate normality*
MVN**::mvn**(dd, mvnTest = "hz", multivariatePlot = TRUE, showOutliers = TRUE, showNewData = TRUE)

## $multivariateNormality
## Test HZ p value MVN
## 1 Henze-Zirkler 2.133026 0 NO
##
## $univariateNormality
## Test Variable Statistic p value Normality
## 1 Shapiro-Wilk RTF_RC1 0.8506 <0.001 NO
## 2 Shapiro-Wilk RTF_RC2 0.8242 <0.001 NO
## 3 Shapiro-Wilk RTF_RC3 0.8267 <0.001 NO
## 4 Shapiro-Wilk RTF_RC4 0.8522 <0.001 NO
## 5 Shapiro-Wilk RTF_RC5 0.8259 <0.001 NO
## 6 Shapiro-Wilk RTF_RC6 0.8083 <0.001 NO
## 7 Shapiro-Wilk RTF_RC7 0.7915 <0.001 NO
## 8 Shapiro-Wilk RTF_A1 0.8077 <0.001 NO
## 9 Shapiro-Wilk RTF_A2 0.7968 <0.001 NO
## 10 Shapiro-Wilk RTF_A3 0.7746 <0.001 NO
## 11 Shapiro-Wilk RTF_A4 0.7921 <0.001 NO
## 12 Shapiro-Wilk RTF_A5 0.7722 <0.001 NO
## 13 Shapiro-Wilk RTF_A6 0.7929 <0.001 NO
## 14 Shapiro-Wilk RTF_A7 0.7757 <0.001 NO
##
## $Descriptives
## n Mean Std.Dev Median Min Max 25th 75th Skew Kurtosis
## RTF_RC1 503 1.2962227 1.2957660 1 0 5 0 2 0.9475219 0.2769938
## RTF_RC2 503 1.1689861 1.2754471 1 0 5 0 2 1.0564568 0.3756429
## RTF_RC3 503 1.1312127 1.2266464 1 0 5 0 2 1.0227841 0.3363873
## RTF_RC4 503 1.1948310 1.1799340 1 0 5 0 2 0.9049598 0.3101344
## RTF_RC5 503 1.3061630 1.4302167 1 0 5 0 2 1.0236952 0.1602888
## RTF_RC6 503 1.0695825 1.2292619 1 0 5 0 2 1.1521407 0.7467903
## RTF_RC7 503 1.1610338 1.3625295 1 0 5 0 2 1.2831953 0.9109107
## RTF_A1 503 1.1192843 1.2895867 1 0 5 0 2 1.1683883 0.7100887
## RTF_A2 503 0.8508946 0.9653531 1 0 5 0 1 1.1357351 1.0996833
## RTF_A3 503 0.8449304 1.0370666 1 0 5 0 1 1.3707658 1.9135375
## RTF_A4 503 0.8926441 1.0160101 1 0 5 0 1 1.2842786 1.5878133
## RTF_A5 503 0.8369781 1.0281227 1 0 5 0 1 1.3604763 1.6261856
## RTF_A6 503 0.9204771 1.0940067 1 0 5 0 1 1.1502864 0.7558339
## RTF_A7 503 0.8866799 1.0735323 1 0 5 0 1 1.4307971 2.0591964
##
## $multivariateOutliers
## NULL
##
## $newData
## NULL

*# one-factor model*
RTF.model1 <- 'RTF =~ RTF_RC1 + RTF_RC2 + RTF_RC3 + RTF_RC4 + RTF_RC5 +

RTF_RC6 + RTF_RC7 + RTF_A1 + RTF_A2 + RTF_A3 + RTF_A4 + RTF_A5 + RTF_A6 + RTF_A7'
m1 <- lavaan**::cfa**(RTF.model1, data = dd, estimator = "MLM")
**summary**(m1, standardized = TRUE, fit.measure = TRUE)

## lavaan 0.6-6 ended normally after 46 iterations
##
## Estimator ML
## Optimization method NLMINB
## Number of free parameters 28
##
## Used Total
## Number of observations 503 508
##
## Model Test User Model:
## Standard Robust
## Test Statistic 165.845 121.608
## Degrees of freedom 77 77
## P-value (Chi-square) 0.000 0.001
## Scaling correction factor 1.364
## Satorra-Bentler correction
##
## Model Test Baseline Model:
##
## Test statistic 771.086 567.303
## Degrees of freedom 91 91
## P-value 0.000 0.000
## Scaling correction factor 1.359
##
## User Model versus Baseline Model:
##
## Comparative Fit Index (CFI) 0.869 0.906
## Tucker-Lewis Index (TLI) 0.846 0.889
##
## Robust Comparative Fit Index (CFI) 0.906
## Robust Tucker-Lewis Index (TLI) 0.889
##
## Loglikelihood and Information Criteria:
##
## Loglikelihood user model (H0) -10791.954 -10791.954
## Loglikelihood unrestricted model (H1) -10709.032 -10709.032
##
## Akaike (AIC) 21639.909 21639.909
## Bayesian (BIC) 21758.085 21758.085
## Sample-size adjusted Bayesian (BIC) 21669.211 21669.211
##
## Root Mean Square Error of Approximation:
##
## RMSEA 0.048 0.034
## 90 Percent confidence interval - lower 0.038 0.024
## 90 Percent confidence interval - upper 0.058 0.043
## P-value RMSEA <= 0.05 0.621 0.998
##
## Robust RMSEA 0.040
## 90 Percent confidence interval - lower 0.026
## 90 Percent confidence interval - upper 0.053
##
## Standardized Root Mean Square Residual:
##
## SRMR 0.048 0.048
##
## Parameter Estimates:
##
## Standard errors Robust.sem
## Information Expected
## Information saturated (h1) model Structured
##
## Latent Variables:
## Estimate Std.Err z-value P(>|z|) Std.lv Std.all
## RTF =~
## RTF_RC1 1.000 0.401 0.310
## RTF_RC2 1.411 0.267 5.278 0.000 0.566 0.444
## RTF_RC3 1.277 0.319 4.005 0.000 0.512 0.418
## RTF_RC4 1.109 0.247 4.499 0.000 0.445 0.377
## RTF_RC5 1.532 0.353 4.341 0.000 0.614 0.430
## RTF_RC6 1.477 0.317 4.657 0.000 0.592 0.482
## RTF_RC7 1.093 0.271 4.033 0.000 0.438 0.322
## RTF_A1 1.160 0.284 4.088 0.000 0.465 0.361
## RTF_A2 0.900 0.230 3.906 0.000 0.361 0.374
## RTF_A3 1.358 0.295 4.597 0.000 0.545 0.526
## RTF_A4 0.675 0.166 4.065 0.000 0.271 0.267
## RTF_A5 1.061 0.244 4.352 0.000 0.426 0.414
## RTF_A6 1.147 0.273 4.205 0.000 0.460 0.421
## RTF_A7 1.212 0.253 4.793 0.000 0.486 0.453
##
## Variances:
## Estimate Std.Err z-value P(>|z|) Std.lv Std.all
## .RTF_RC1 1.515 0.118 12.837 0.000 1.515 0.904
## .RTF_RC2 1.303 0.101 12.951 0.000 1.303 0.803
## .RTF_RC3 1.240 0.086 14.458 0.000 1.240 0.825
## .RTF_RC4 1.192 0.092 12.971 0.000 1.192 0.858
## .RTF_RC5 1.664 0.119 13.942 0.000 1.664 0.815
## .RTF_RC6 1.158 0.091 12.711 0.000 1.158 0.768
## .RTF_RC7 1.661 0.138 12.065 0.000 1.661 0.896
## .RTF_A1 1.443 0.111 13.028 0.000 1.443 0.870
## .RTF_A2 0.800 0.064 12.478 0.000 0.800 0.860
## .RTF_A3 0.777 0.067 11.548 0.000 0.777 0.724
## .RTF_A4 0.957 0.079 12.066 0.000 0.957 0.929
## .RTF_A5 0.874 0.080 10.882 0.000 0.874 0.828
## .RTF_A6 0.983 0.081 12.092 0.000 0.983 0.823
## .RTF_A7 0.914 0.083 10.979 0.000 0.914 0.795
## RTF 0.161 0.057 2.809 0.005 1.000 1.000

**fitMeasures**(m1, **c**("rmsea.robust", "rmsea.ci.lower.robust", "rmsea.ci.upper.robust", "cfi.robust", "tli.robust", "ifi.scaled"))

## rmsea.robust rmsea.ci.lower.robust rmsea.ci.upper.robust
## 0.040 0.026 0.053
## cfi.robust tli.robust ifi.scaled
## 0.906 0.889 0.909

semTools**::reliability**(m1)

## RTF
## alpha 0.7229750
## omega 0.7243891
## omega2 0.7243891
## omega3 0.7231290
## avevar 0.1628847

semPlot**::semPaths**(m1, "std", rotation = 2)


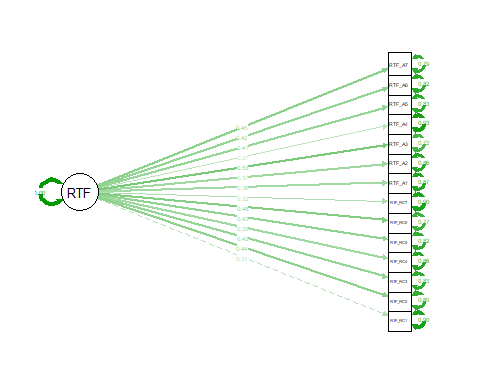


*# two-factor model*
RTF.model2 <- 'risky_choice =~ RTF_RC1 + RTF_RC2 + RTF_RC3 + RTF_RC4 +

RTF_RC5 + RTF_RC6 + RTF_RC7
 attribute =~ RTF_A1 + RTF_A2 + RTF_A3 + RTF_A4 + RTF_A5 + RTF_A6 + RTF_A7
 risky_choice ~~ attribute'

m2 <- lavaan**::cfa**(RTF.model2, data = dd, estimator = "MLM")
**summary**(m2, standardized = TRUE, fit.measure = TRUE)

## lavaan 0.6-6 ended normally after 45 iterations
##
## Estimator ML
## Optimization method NLMINB
## Number of free parameters 29
##
## Used Total
## Number of observations 503 508
##
## Model Test User Model:
## Standard Robust
## Test Statistic 108.606 78.810
## Degrees of freedom 76 76
## P-value (Chi-square) 0.008 0.390
## Scaling correction factor 1.378
## Satorra-Bentler correction
##
## Model Test Baseline Model:
##
## Test statistic 771.086 567.303
## Degrees of freedom 91 91
## P-value 0.000 0.000
## Scaling correction factor 1.359
##
## User Model versus Baseline Model:
##
## Comparative Fit Index (CFI) 0.952 0.994
## Tucker-Lewis Index (TLI) 0.943 0.993
##
## Robust Comparative Fit Index (CFI) 0.994
## Robust Tucker-Lewis Index (TLI) 0.993
##
## Loglikelihood and Information Criteria:
##
## Loglikelihood user model (H0) -10763.335 -10763.335
## Loglikelihood unrestricted model (H1) -10709.032 -10709.032
##
## Akaike (AIC) 21584.669 21584.669
## Bayesian (BIC) 21707.067 21707.067
## Sample-size adjusted Bayesian (BIC) 21615.018 21615.018
##
## Root Mean Square Error of Approximation:
##
## RMSEA 0.029 0.009
## 90 Percent confidence interval - lower 0.015 0.000
## 90 Percent confidence interval - upper 0.041 0.025
## P-value RMSEA <= 0.05 0.999 1.000
##
## Robust RMSEA 0.010
## 90 Percent confidence interval - lower 0.000
## 90 Percent confidence interval - upper 0.032
##
## Standardized Root Mean Square Residual:
##
## SRMR 0.038 0.038
##
## Parameter Estimates:
##
## Standard errors Robust.sem
## Information Expected
## Information saturated (h1) model Structured
##
## Latent Variables:
## Estimate Std.Err z-value P(>|z|) Std.lv Std.all
## risky_choice =~
## RTF_RC1 1.000 0.449 0.347
## RTF_RC2 1.468 0.277 5.302 0.000 0.659 0.518
## RTF_RC3 1.370 0.310 4.413 0.000 0.616 0.502
## RTF_RC4 1.069 0.225 4.759 0.000 0.480 0.407
## RTF_RC5 1.466 0.324 4.528 0.000 0.659 0.461
## RTF_RC6 1.388 0.291 4.778 0.000 0.624 0.508
## RTF_RC7 1.037 0.251 4.136 0.000 0.466 0.342
## attribute =~
## RTF_A1 1.000 0.544 0.422
## RTF_A2 0.660 0.133 4.968 0.000 0.359 0.372
## RTF_A3 1.120 0.161 6.939 0.000 0.609 0.588
## RTF_A4 0.500 0.135 3.704 0.000 0.272 0.268
## RTF_A5 0.905 0.134 6.771 0.000 0.493 0.480
## RTF_A6 0.940 0.162 5.819 0.000 0.512 0.468
## RTF_A7 0.956 0.198 4.829 0.000 0.520 0.485
##
## Covariances:
## Estimate Std.Err z-value P(>|z|) Std.lv Std.all
## risky_choice ~~
## attribute 0.162 0.037 4.334 0.000 0.661 0.661
##
## Variances:
## Estimate Std.Err z-value P(>|z|) Std.lv Std.all
## .RTF_RC1 1.474 0.119 12.378 0.000 1.474 0.880
## .RTF_RC2 1.189 0.099 12.022 0.000 1.189 0.732
## .RTF_RC3 1.123 0.090 12.489 0.000 1.123 0.748
## .RTF_RC4 1.159 0.094 12.391 0.000 1.159 0.834
## .RTF_RC5 1.608 0.121 13.289 0.000 1.608 0.787
## .RTF_RC6 1.119 0.094 11.874 0.000 1.119 0.742
## .RTF_RC7 1.636 0.136 11.984 0.000 1.636 0.883
## .RTF_A1 1.364 0.112 12.211 0.000 1.364 0.822
## .RTF_A2 0.801 0.064 12.432 0.000 0.801 0.861
## .RTF_A3 0.702 0.069 10.237 0.000 0.702 0.654
## .RTF_A4 0.956 0.082 11.692 0.000 0.956 0.928
## .RTF_A5 0.812 0.082 9.934 0.000 0.812 0.770
## .RTF_A6 0.933 0.082 11.344 0.000 0.933 0.781
## .RTF_A7 0.879 0.093 9.506 0.000 0.879 0.765
## risky_choice 0.202 0.068 2.959 0.003 1.000 1.000
## attribute 0.296 0.082 3.596 0.000 1.000 1.000

**fitMeasures**(m2, **c**("rmsea.robust", "rmsea.ci.lower.robust", "rmsea.ci.upper.robust", "cfi.robust", "tli.robust", "ifi.scaled"))

## rmsea.robust rmsea.ci.lower.robust rmsea.ci.upper.robust
## 0.010 0.000 0.032
## cfi.robust tli.robust ifi.scaled
## 0.994 0.993 0.994

semTools**::reliability**(m2)

## risky_choice attribute
## alpha 0.6245487 0.6226999
## omega 0.6267071 0.6294174
## omega2 0.6267071 0.6294174
## omega3 0.6262774 0.6308966
## avevar 0.1972048 0.2033290

semPlot**::semPaths**(m2, "std", rotation = 2)


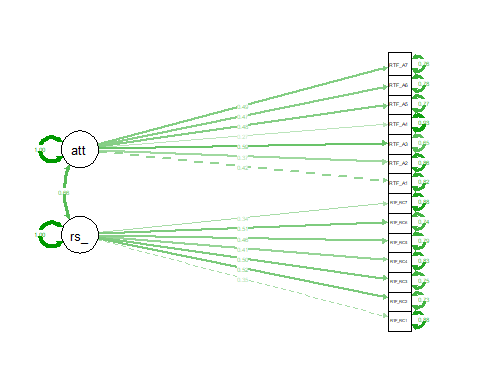


*# compare the models*
**anova**(m1, m2)

## Scaled Chi-Squared Difference Test (method = "satorra.bentler.2001")
##
## lavaan NOTE:
## The "Chisq" column contains standard test statistics, not the
## robust test that should be reported per model. A robust difference
## test is a function of two standard (not robust) statistics.
##
## Df AIC BIC Chisq Chisq diff Df diff Pr(>Chisq)
## m2 76 21585 21707 108.61
## m1 77 21640 21758 165.84 206.26 1 < 2.2e-16 ***
## ---
## Signif. codes: 0 '***' 0.001 '**' 0.01 '*' 0.05 '.' 0.1 ' ' 1

**Data US**

Note: RTF.model1 and RTF.model2 are missing the variable rc2abs, because a typo changed the meaning of this item (see Bruine de Bruin et al., 2007).

*# read the dataset*
path <- "data_us.sav"
data <- foreign**::read.spss**(path, use.value.labels = T, to.data.frame = T)

## re-encoding from UTF-8

*# only framing difference scores*
d <- data **%>%** dplyr**::select**(**ends_with**("abs"))

*# Multivariate normality*
MVN**::mvn**(d, mvnTest = "hz", multivariatePlot = TRUE, showOutliers = TRUE, showNewData = TRUE)

## $multivariateNormality
## Test HZ p value MVN
## 1 Henze-Zirkler 1.397796 0 NO
##
## $univariateNormality
## Test Variable Statistic p value Normality
## 1 Shapiro-Wilk rc1abs 0.8839 <0.001 NO
## 2 Shapiro-Wilk rc3abs 0.8673 <0.001 NO
## 3 Shapiro-Wilk rc4abs 0.8595 <0.001 NO
## 4 Shapiro-Wilk rc5abs 0.8571 <0.001 NO
## 5 Shapiro-Wilk rc6abs 0.8180 <0.001 NO
## 6 Shapiro-Wilk rc7abs 0.8238 <0.001 NO
## 7 Shapiro-Wilk a1abs 0.7322 <0.001 NO
## 8 Shapiro-Wilk a2abs 0.7604 <0.001 NO
## 9 Shapiro-Wilk a3abs 0.7914 <0.001 NO
## 10 Shapiro-Wilk a4abs 0.8013 <0.001 NO
## 11 Shapiro-Wilk a5abs 0.7278 <0.001 NO
## 12 Shapiro-Wilk a6abs 0.7931 <0.001 NO
## 13 Shapiro-Wilk a7abs 0.7877 <0.001 NO
##
## $Descriptives
## n Mean Std.Dev Median Min Max 25th 75th Skew Kurtosis
## rc1abs 278 3.325540 1.462885 4 0 5 2.25 4 -0.7191608 -0.32947203
## rc3abs 278 3.392086 1.454810 4 0 5 2.25 4 -0.8380496 -0.14224629
## rc4abs 278 3.460432 1.485264 4 0 5 3.00 5 -0.8422684 -0.21689770
## rc5abs 278 3.422662 1.537248 4 0 5 3.00 5 -0.8269398 -0.29367263
## rc6abs 278 3.665468 1.464241 4 0 5 3.00 5 -1.0823070 0.27005466
## rc7abs 278 3.602518 1.497686 4 0 5 3.00 5 -1.0316975 0.06798015
## a1abs 278 3.956835 1.443249 5 0 5 4.00 5 -1.4298918 1.05774176
## a2abs 278 4.041367 1.247053 4 0 5 4.00 5 -1.3639445 1.10855005
## a3abs 278 4.066547 1.089860 4 0 5 3.00 5 -1.3327882 1.87736926
## a4abs 278 3.906475 1.210019 4 0 5 3.00 5 -1.3440755 1.60277584
## a5abs 278 4.098921 1.167443 4 0 5 4.00 5 -1.7779057 3.15228332
## a6abs 278 3.931655 1.295927 4 0 5 3.00 5 -1.2061579 0.78599829
## a7abs 278 3.893885 1.304144 4 0 5 3.00 5 -1.3712752 1.44593910
##
## $multivariateOutliers
## NULL
##
## $newData
## NULL

*# one-factor model*
RTF.model1 <- 'RTF =~ rc1abs + rc3abs + rc4abs + rc5abs + rc6abs + rc7abs + a1abs + a2abs + a3abs + a4abs + a5abs + a6abs + a7abs'

m1 <- lavaan**::cfa**(RTF.model1, data = d, estimator = "MLM")
**summary**(m1, standardized = TRUE, fit.measure = TRUE)

## lavaan 0.6-6 ended normally after 75 iterations
##
## Estimator ML
## Optimization method NLMINB
## Number of free parameters 26
##
## Used Total
## Number of observations 278 360
##
## Model Test User Model:
## Standard Robust
## Test Statistic 129.856 90.167
## Degrees of freedom 65 65
## P-value (Chi-square) 0.000 0.021
## Scaling correction factor 1.440
## Satorra-Bentler correction
##
## Model Test Baseline Model:
##
## Test statistic 321.606 216.509
## Degrees of freedom 78 78
## P-value 0.000 0.000
## Scaling correction factor 1.485
##
## User Model versus Baseline Model:
##
## Comparative Fit Index (CFI) 0.734 0.818
## Tucker-Lewis Index (TLI) 0.681 0.782
##
## Robust Comparative Fit Index (CFI) 0.824
## Robust Tucker-Lewis Index (TLI) 0.789
##
## Loglikelihood and Information Criteria:
##
## Loglikelihood user model (H0) -6112.661 -6112.661
## Loglikelihood unrestricted model (H1) -6047.733 -6047.733
##
## Akaike (AIC) 12277.321 12277.321
## Bayesian (BIC) 12371.639 12371.639
## Sample-size adjusted Bayesian (BIC) 12289.196 12289.196
##
## Root Mean Square Error of Approximation:
##
## RMSEA 0.060 0.037
## 90 Percent confidence interval - lower 0.045 0.020
## 90 Percent confidence interval - upper 0.075 0.052
## P-value RMSEA <= 0.05 0.134 0.920
##
## Robust RMSEA 0.045
## 90 Percent confidence interval - lower 0.018
## 90 Percent confidence interval - upper 0.066
##
## Standardized Root Mean Square Residual:
##
## SRMR 0.062 0.062
##
## Parameter Estimates:
##
## Standard errors Robust.sem
## Information Expected
## Information saturated (h1) model Structured
##
## Latent Variables:
## Estimate Std.Err z-value P(>|z|) Std.lv Std.all
## RTF =~
## rc1abs 1.000 0.272 0.186
## rc3abs 1.299 0.721 1.801 0.072 0.353 0.243
## rc4abs 1.707 0.972 1.757 0.079 0.464 0.313
## rc5abs 1.206 0.598 2.018 0.044 0.328 0.214
## rc6abs 1.541 0.863 1.786 0.074 0.419 0.287
## rc7abs 1.650 0.887 1.861 0.063 0.449 0.300
## a1abs 2.949 1.448 2.036 0.042 0.801 0.556
## a2abs 1.954 1.156 1.691 0.091 0.531 0.427
## a3abs 1.600 0.830 1.929 0.054 0.435 0.400
## a4abs 0.907 0.568 1.597 0.110 0.246 0.204
## a5abs 2.010 1.019 1.973 0.048 0.546 0.469
## a6abs 1.563 0.770 2.030 0.042 0.425 0.328
## a7abs 2.062 1.009 2.043 0.041 0.560 0.430
##
## Variances:
## Estimate Std.Err z-value P(>|z|) Std.lv Std.all
## .rc1abs 2.058 0.156 13.158 0.000 2.058 0.965
## .rc3abs 1.984 0.173 11.486 0.000 1.984 0.941
## .rc4abs 1.983 0.171 11.605 0.000 1.983 0.902
## .rc5abs 2.247 0.179 12.558 0.000 2.247 0.954
## .rc6abs 1.961 0.184 10.634 0.000 1.961 0.918
## .rc7abs 2.034 0.190 10.693 0.000 2.034 0.910
## .a1abs 1.433 0.162 8.866 0.000 1.433 0.691
## .a2abs 1.267 0.153 8.309 0.000 1.267 0.818
## .a3abs 0.994 0.129 7.737 0.000 0.994 0.840
## .a4abs 1.398 0.152 9.201 0.000 1.398 0.958
## .a5abs 1.060 0.161 6.572 0.000 1.060 0.780
## .a6abs 1.493 0.161 9.278 0.000 1.493 0.892
## .a7abs 1.381 0.176 7.841 0.000 1.381 0.815
## RTF 0.074 0.072 1.028 0.304 1.000 1.000

**fitMeasures**(m1, **c**("rmsea.robust", "rmsea.ci.lower.robust", "rmsea.ci.upper.robust", "cfi.robust", "tli.robust", "ifi.scaled"))

## rmsea.robust rmsea.ci.lower.robust rmsea.ci.upper.robust
## 0.045 0.018 0.066
## cfi.robust tli.robust ifi.scaled
## 0.824 0.789 0.834

semTools**::reliability**(m1)

## RTF
## alpha 0.6191182
## omega 0.6147322
## omega2 0.6147322
## omega3 0.6026480
## avevar 0.1185789

semPlot**::semPaths**(m1, "std", rotation = 2)


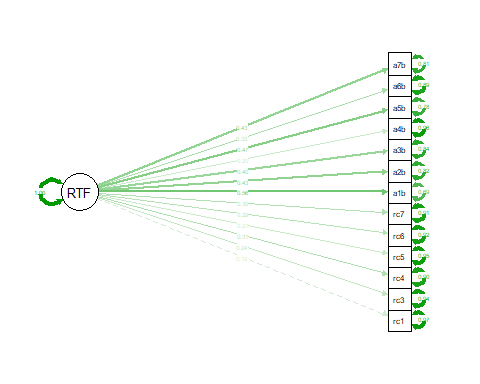


*# two-factor model*
RTF.model2 <- 'risky_choice =~ rc1abs + rc3abs + rc4abs + rc5abs + rc6abs + rc7abs
 attribute =~ a1abs + a2abs + a3abs + a4abs + a5abs + a6abs + a7abs
 risky_choice ~~ attribute'

m2 <- lavaan**::cfa**(RTF.model2, data = d, estimator = "MLR")
**summary**(m2, standardized = TRUE, fit.measure = TRUE)

## lavaan 0.6-6 ended normally after 52 iterations
##
## Estimator ML
## Optimization method NLMINB
## Number of free parameters 27
##
## Used Total
## Number of observations 278 360
##
## Model Test User Model:
## Standard Robust
## Test Statistic 113.736 87.708
## Degrees of freedom 64 64
## P-value (Chi-square) 0.000 0.026
## Scaling correction factor 1.297
## Yuan-Bentler correction (Mplus variant)
##
## Model Test Baseline Model:
##
## Test statistic 321.606 235.256
## Degrees of freedom 78 78
## P-value 0.000 0.000
## Scaling correction factor 1.367
##
## User Model versus Baseline Model:
##
## Comparative Fit Index (CFI) 0.796 0.849
## Tucker-Lewis Index (TLI) 0.751 0.816
##
## Robust Comparative Fit Index (CFI) 0.857
## Robust Tucker-Lewis Index (TLI) 0.826
##
## Loglikelihood and Information Criteria:
##
## Loglikelihood user model (H0) -6104.600 -6104.600
## Scaling correction factor 1.556
## for the MLR correction
## Loglikelihood unrestricted model (H1) -6047.733 -6047.733
## Scaling correction factor 1.374
## for the MLR correction
##
## Akaike (AIC) 12263.201 12263.201
## Bayesian (BIC) 12361.146 12361.146
## Sample-size adjusted Bayesian (BIC) 12275.533 12275.533
##
## Root Mean Square Error of Approximation:
##
## RMSEA 0.053 0.037
## 90 Percent confidence interval - lower 0.037 0.017
## 90 Percent confidence interval - upper 0.068 0.052
## P-value RMSEA <= 0.05 0.365 0.919
##
## Robust RMSEA 0.042
## 90 Percent confidence interval - lower 0.015
## 90 Percent confidence interval - upper 0.062
##
## Standardized Root Mean Square Residual:
##
## SRMR 0.061 0.061
##
## Parameter Estimates:
##
## Standard errors Sandwich
## Information bread Observed
## Observed information based on Hessian
##
## Latent Variables:
## Estimate Std.Err z-value P(>|z|) Std.lv Std.all
## risky_choice =~
## rc1abs 1.000 0.484 0.331
## rc3abs 0.895 0.450 1.991 0.046 0.433 0.298
## rc4abs 1.626 0.700 2.323 0.020 0.787 0.531
## rc5abs 0.499 0.417 1.198 0.231 0.242 0.157
## rc6abs 1.443 0.545 2.649 0.008 0.698 0.478
## rc7abs 0.687 0.501 1.371 0.170 0.333 0.222
## attribute =~
## a1abs 1.000 0.854 0.593
## a2abs 0.623 0.218 2.854 0.004 0.532 0.428
## a3abs 0.482 0.167 2.890 0.004 0.412 0.379
## a4abs 0.310 0.139 2.230 0.026 0.265 0.219
## a5abs 0.697 0.179 3.896 0.000 0.596 0.511
## a6abs 0.492 0.159 3.094 0.002 0.420 0.325
## a7abs 0.649 0.141 4.600 0.000 0.554 0.426
##
## Covariances:
## Estimate Std.Err z-value P(>|z|) Std.lv Std.all
## risky_choice ~~
## attribute 0.218 0.102 2.142 0.032 0.527 0.527
##
## Variances:
## Estimate Std.Err z-value P(>|z|) Std.lv Std.all
## .rc1abs 1.898 0.174 10.890 0.000 1.898 0.890
## .rc3abs 1.921 0.196 9.799 0.000 1.921 0.911
## .rc4abs 1.579 0.314 5.030 0.000 1.579 0.718
## .rc5abs 2.296 0.195 11.788 0.000 2.296 0.975
## .rc6abs 1.649 0.265 6.221 0.000 1.649 0.772
## .rc7abs 2.124 0.220 9.677 0.000 2.124 0.951
## .a1abs 1.346 0.196 6.861 0.000 1.346 0.649
## .a2abs 1.266 0.161 7.849 0.000 1.266 0.817
## .a3abs 1.014 0.137 7.424 0.000 1.014 0.857
## .a4abs 1.389 0.154 9.016 0.000 1.389 0.952
## .a5abs 1.003 0.157 6.373 0.000 1.003 0.739
## .a6abs 1.497 0.164 9.106 0.000 1.497 0.895
## .a7abs 1.388 0.190 7.292 0.000 1.388 0.819
## risky_choice 0.234 0.141 1.657 0.098 1.000 1.000
## attribute 0.729 0.266 2.739 0.006 1.000 1.000

**semTable**(m2, file = "table.csv", type = "csv", print.results = TRUE)

## ,Model,
##
## ,Estimate,Std. Err.,z,p,
##
## ,Factor Loadings,
## risky_choice,
##
## rc1abs,1.00+,,,,
##
## rc3abs,0.90,0.45,1.99,.046,
##
## rc4abs,1.63,0.70,2.32,.020,
##
## rc5abs,0.50,0.42,1.20,.231,
##
## rc6abs,1.44,0.54,2.65,.008,
##
## rc7abs,0.69,0.50,1.37,.170,
##
## attribute,
##
## a1abs,1.00+,,,,
##
## a2abs,0.62,0.22,2.85,.004,
##
## a3abs,0.48,0.17,2.89,.004,
##
## a4abs,0.31,0.14,2.23,.026,
##
## a5abs,0.70,0.18,3.90,.000,
##
## a6abs,0.49,0.16,3.09,.002,
##
## a7abs,0.65,0.14,4.60,.000,
##
## ,Residual Variances,
##
## rc1abs,1.90,0.17,10.89,.000,
##
## rc3abs,1.92,0.20,9.80,.000,
##
## rc4abs,1.58,0.31,5.03,.000,
##
## rc5abs,2.30,0.19,11.79,.000,
##
## rc6abs,1.65,0.27,6.22,.000,
##
## rc7abs,2.12,0.22,9.68,.000,
##
## a1abs,1.35,0.20,6.86,.000,
##
## a2abs,1.27,0.16,7.85,.000,
##
## a3abs,1.01,0.14,7.42,.000,
##
## a4abs,1.39,0.15,9.02,.000,
##
## a5abs,1.00,0.16,6.37,.000,
##
## a6abs,1.50,0.16,9.11,.000,
##
## a7abs,1.39,0.19,7.29,.000,
##
## ,Latent Variances,
##
## risky.choice,0.23,0.14,1.66,.098,
##
## attribute,0.73,0.27,2.74,.006,
##
## ,Latent Covariances,
##
## risky.choice w/attribute,0.22,0.10,2.14,.032,
##
## ,Fit Indices,
##
## chi^2,113.74,,,,
##
## CFI,0.80,,,,
##
## TLI,0.75,,,,
##
## RMSEA,0.05,,,,
##
## Scaled chi^2,87.71(64),,,.026,
##
## +Fixed parameter,
##
##
##
##

**fitMeasures**(m2, **c**("rmsea.robust", "rmsea.ci.lower.robust", "rmsea.ci.upper.robust", "cfi.robust", "tli.robust", "ifi.scaled"))

## rmsea.robust rmsea.ci.lower.robust rmsea.ci.upper.robust
## 0.042 0.015 0.062
## cfi.robust tli.robust ifi.scaled
## 0.857 0.826 0.862

semTools**::reliability**(m2)

## risky_choice attribute
## alpha 0.4183079 0.5813306
## omega 0.4357377 0.5971879
## omega2 0.4357377 0.5971879
## omega3 0.4381960 0.6023555
## avevar 0.1288967 0.1901840

semPlot**::semPaths**(m2, "std", rotation = 2)


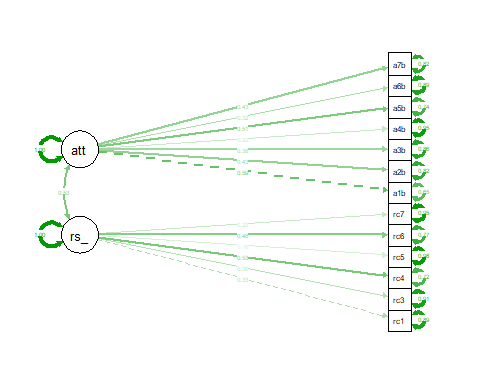


*# compare the models*
**anova**(m1, m2)

## Scaled Chi-Squared Difference Test (method = "satorra.bentler.2001")
##
## lavaan NOTE:
## The "Chisq" column contains standard test statistics, not the
## robust test that should be reported per model. A robust difference
## test is a function of two standard (not robust) statistics.
##
## Df AIC BIC Chisq Chisq diff Df diff Pr(>Chisq)
## m2 64 12263 12361 113.74
## m1 65 12277 12372 129.86 1.518 1 0.2179

**Power Analyses**

For the a priori power analyses to detect model fit and misfit, the RMSEA cut-off value for acceptable fit was set to .08. The true RMSEA values were derived from reanalyzing previously collected data using the one-factor model (RMSEA = .04, Bavolar, 2013; Bruine de Bruin et al., 2007) and the two-factor model (RMSEA = .01, Bavolar, 2013).

*## one-factor model*
semTools**::findRMSEAsamplesize**(rmsea0 = .04, rmseaA = .08, df = 77,

power = .95)

## [1] 166

*## two-factor model*
semTools**::findRMSEAsamplesize**(rmsea0 = .01, rmseaA = .08, df = 76,

power = .95)

## [1] 109

With a sample size of 250 participants per site, this yields a power of more than 95% to detect model fit of both the one- and two-factor model.

**Session Info**

**sessionInfo**()

## R version 4.0.2 (2020-06-22)
## Platform: x86_64-w64-mingw32/x64 (64-bit)
## Running under: Windows 10 x64 (build 19041)
##
## Matrix products: default
##
## locale:
## [1] LC_COLLATE=German_Germany.1252 LC_CTYPE=German_Germany.1252
## [3] LC_MONETARY=German_Germany.1252 LC_NUMERIC=C
## [5] LC_TIME=German_Germany.1252
##
## attached base packages:
## [1] stats graphics grDevices utils datasets methods base
##
## other attached packages:
## [1] semTable_1.8 MVN_5.8 reshape2_1.4.4 semPlot_1.1.2
## [5] semTools_0.5-3 psych_1.9.12.31 lavaan_0.6-6 forcats_0.5.0
## [9] stringr_1.4.0 dplyr_1.0.2 purrr_0.3.4 readr_1.3.1
## [13] tidyr_1.1.2 tibble_3.0.3 ggplot2_3.3.2 tidyverse_1.3.0
##
## loaded via a namespace (and not attached):
## [1] tidyselect_1.1.0 lme4_1.1-26 htmlwidgets_1.5.1
## [4] grid_4.0.2 ranger_0.12.1 munsell_0.5.0
## [7] statmod_1.4.34 sROC_0.1-2 withr_2.2.0
## [10] colorspace_1.4-1 energy_1.7-7 OpenMx_2.17.4
## [13] knitr_1.30 rstudioapi_0.11 stats4_4.0.2
## [16] robustbase_0.93-6 vcd_1.4-7 VIM_6.0.0
## [19] huge_1.3.4.1 mi_1.0 emmeans_1.4.8
## [22] cvTools_0.3.2 mnormt_2.0.1 coda_0.19-3
## [25] vctrs_0.3.4 generics_0.0.2 xfun_0.18
## [28] diptest_0.75-7 R6_2.4.1 robCompositions_2.2.1
## [31] arm_1.11-1 mvoutlier_2.0.9 flexmix_2.3-15
## [34] reshape_0.8.8 assertthat_0.2.1 scales_1.1.1
## [37] nnet_7.3-14 gtable_0.3.0 rlang_0.4.7
## [40] splines_4.0.2 acepack_1.4.1 broom_0.7.0
## [43] checkmate_2.0.0 yaml_2.2.1 abind_1.4-5
## [46] modelr_0.1.8 d3Network_0.5.2.1 backports_1.1.9
## [49] Hmisc_4.4-0 tools_4.0.2 zCompositions_1.3.4
## [52] ellipsis_0.3.1 kableExtra_1.1.0 RColorBrewer_1.1-2
## [55] Rsolnp_1.16 stationery_0.98.30 Rcpp_1.0.5
## [58] plyr_1.8.6 base64enc_0.1-3 rockchalk_1.8.144
## [61] rpart_4.1-15 pbapply_1.4-2 zoo_1.8-8
## [64] qgraph_1.6.5 haven_2.3.1 cluster_2.1.0
## [67] fs_1.4.2 magrittr_1.5 data.table_1.12.8
## [70] openxlsx_4.1.5 lmtest_0.9-37 reprex_0.3.0
## [73] truncnorm_1.0-8 tmvnsim_1.0-2 mvtnorm_1.1-1
## [76] matrixcalc_1.0-3 whisker_0.4 hms_0.5.3
## [79] evaluate_0.14 xtable_1.8-4 XML_3.99-0.4
## [82] rio_0.5.16 jpeg_0.1-8.1 mclust_5.4.6
## [85] readxl_1.3.1 gridExtra_2.3 compiler_4.0.2
## [88] crayon_1.3.4 minqa_1.2.4 htmltools_0.5.0
## [91] corpcor_1.6.9 pcaPP_1.9-73 Formula_1.2-3
## [94] rrcov_1.5-2 lubridate_1.7.9 DBI_1.1.0
## [97] kutils_1.70 dbplyr_1.4.4 MASS_7.3-51.6
## [100] fpc_2.2-7 boot_1.3-25 Matrix_1.2-18
## [103] car_3.0-8 cli_2.0.2 sgeostat_1.0-27
## [106] parallel_4.0.2 igraph_1.2.5 BDgraph_2.62
## [109] pkgconfig_2.0.3 sem_3.1-11 foreign_0.8-80
## [112] laeken_0.5.1 sp_1.4-2 xml2_1.3.2
## [115] pbivnorm_0.6.0 webshot_0.5.2 estimability_1.3
## [118] rvest_0.3.5 NADA_1.6-1.1 digest_0.6.25
## [121] pls_2.7-2 rmarkdown_2.4 cellranger_1.1.0
## [124] htmlTable_2.0.1 nortest_1.0-4 lisrelToR_0.1.4
## [127] curl_4.3 kernlab_0.9-29 gtools_3.8.2
## [130] modeltools_0.2-23 rjson_0.2.20 nloptr_1.2.2.2
## [133] lifecycle_0.2.0 nlme_3.1-148 glasso_1.11
## [136] jsonlite_1.7.1 carData_3.0-4 viridisLite_0.3.0
## [139] fansi_0.4.1 pillar_1.4.6 lattice_0.20-41
## [142] GGally_2.0.0 httr_1.4.1 DEoptimR_1.0-8
## [145] survival_3.2-3 glue_1.4.2 zip_2.1.1
## [148] fdrtool_1.2.15 png_0.1-7 prabclus_2.3-2
## [151] class_7.3-17 stringi_1.5.3 regsem_1.5.2
## [154] blob_1.2.1 moments_0.14 latticeExtra_0.6-29
## [157] e1071_1.7-3

# Supplement 2: Deviations From Preregistration

| **Section** | **Preregistered** | **Deviation** | **Reason** |
| --- | --- | --- | --- |
| **Data Exclusions** | Exclude participants if they completed the survey faster than 3.5 median absolute deviations of the overall time (Leys et al., 2019) | Participants were only included if they did not complete the survey faster than 10 min. | We deviated from our preregistered criterion because many participants completed the survey faster than 10 min (*n* = 51 of 341 in North America and *n* = 7 of 282 in Bulgaria). Not changing the outlier criterion would have led to a low  median and including many responses with an unreasonably short completion time. |
| **Results** | **-** | We computed inter-item correlations using network analysis and item-total correlations. | We computed inter-item and item-total correlations to get more insights into the structure of the scale. |
| **Results** | We planned to compare the fit of the one- and two-factor model in each site based on the LRT, AIC, and BIC. If at least two of the three criteria point toward one model, this model would be selected. | We did not compare the fit of the one-and two-factor model. | The low inter-item correlations already pointed to problems with the structure of the scale. These low-inter item correlations may have caused overly good model fit (reliability paradox). For these two reasons, it did not make sense to compare the models further. |
| **Results** | We planned to test measurement invariance between sites. | We did not test measurement variance between sites. | Measurement invariance testing was unnecessary because the inter-item correlations already demonstrated problems with the scale in both sites. |
| **Results** | - | We conducted exploratory IRT analyses. | Due to the low reliability, we conducted IRT analyses to get more detailed insights into the reliability of each item. |

#

# Supplement 3: Analyses Including Outliers

# Bulgaria

**Participants**

The analysis including outliers was based on 275 Bulgarian participants. This sample included participants between 18 and 52 years (*M* = 23.22, *SD* = 6.18, 78.9% female). All participants were Bulgarian citizens and residents, and native Bulgarian speakers. They completed high school (*n* = 224), a three-year bachelor’s degree (*n* = 14), a four-year bachelor’s degree (*n* = 24) or a master’s degree (*n* = 13). They were enrolled in a variety of study programs and years, with none (*n* = 1), first (*n* = 88), second (*n* = 70) third (*n* = 47), forth (*n* = 40), and fifth or higher (*n* = 29) year students.

**Inter-Item and Item-Total Correlations**

Inter-item and item-total correlations were small and ranged from *r* = -.05 to *r* = .26 as well as from *r* = .09 to *r* = .30, respectively. Once again, the network plot (Figure 1 S3) shows no clear clustering pattern.

**Figure 1 S3**

*Network Plot of the Bivariate Correlations Between the Items of the Resistance to Framing Scale for Bulgaria (Including Outliers)*


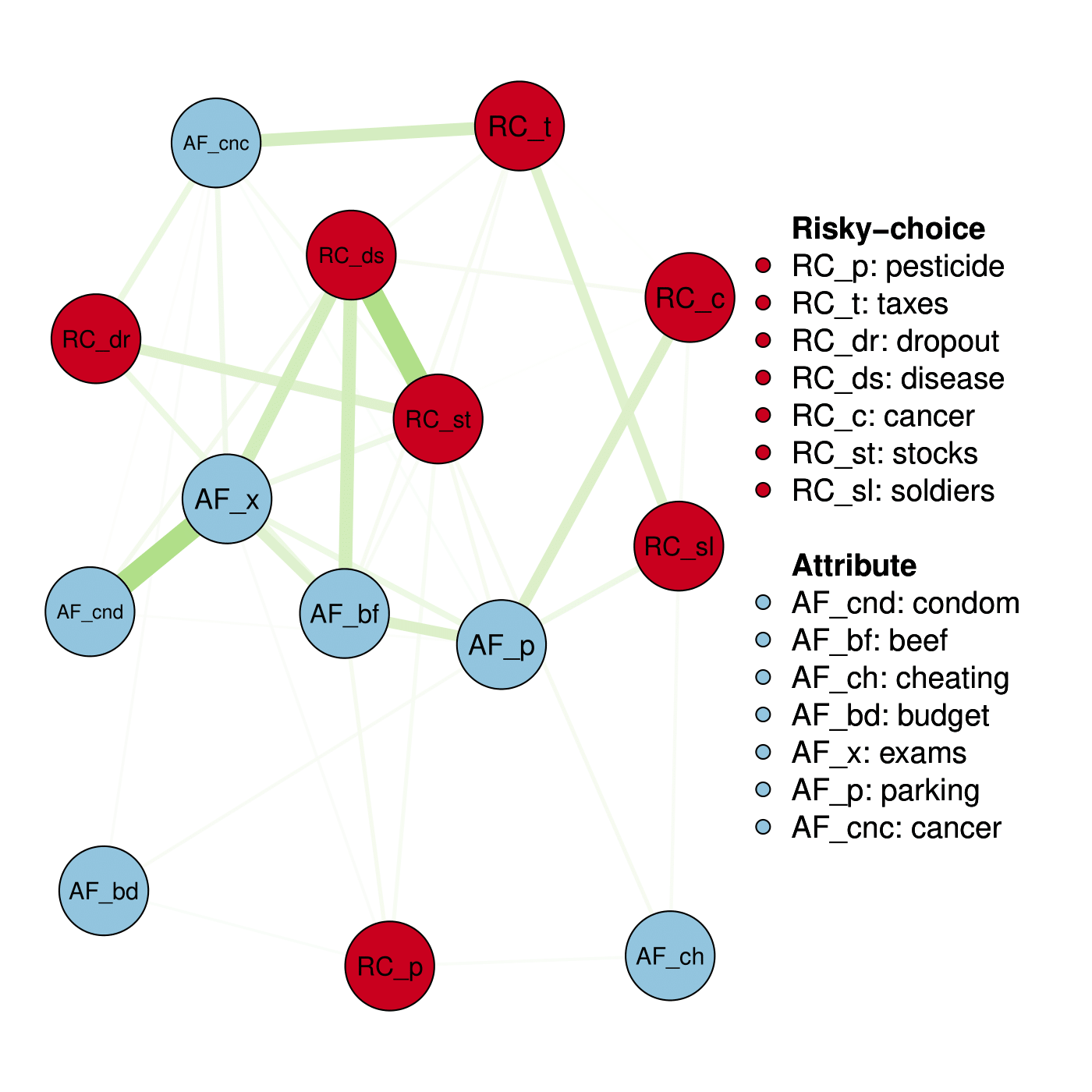


**Assumptions**

As multivariate normality was violated (*HZ* = 1.69, *p* < .001), the models were fit using robust ML estimation.

**One-Factor vs. Two-Factor Model**

The hypothesized one-factor model fit the data acceptably based on our preregistered criteria. The robust χ^2^-test indicated exact fit (χ^2^(77) = 66.81, *p* = .790, scale correction factor = 1.18). The robust RMSEA suggested acceptable fit, as well as the robust incremental fit indices (RMSEA = .00, 90% CI[.00, .03], CFI = 1.00, TLI = 1.15, IFI = 1.11). However, two items (i.e., attribute framing: cheating and budget) did not load significantly onto the RtF factor.

The hypothesized two-factor model fit the data acceptably. The robust χ^2^-test suggested no exact fit (χ^2^(76) = 65.02, *p* = .811, scale correction factor = 1.17). The robust RMSEA indicated excellent fit (RMSEA = .00, 90% CI[.00, .03]). The robust incremental fit indices suggested excellent fit (CFI = 1.00, TLI = 1.16, IFI = 1.12). Again, two of 14 items (i.e., attribute framing: cheating and budget) did not load significantly onto their corresponding factor.

**Reliability**

All scales showed poor reliability with a McDonald’s ω of .41 for both risky-choice and attribute framing and a McDonald’s ω of .55 for the entire Resistance to Framing scale.

**Conclusion**

The results are robust to outlier exclusions. However, the reliability of the risky-choice and attribute framing subscales was higher when including rather than excluding outliers.

# North America

**Participants**

The analysis including outliers was based on 290 North American participants. This sample included participants between 18 and 49 years (*M* = 21.90, *SD* = 4.28, 43.4% female). Most participants were US citizens and residents (*n* = 218), and all participants were English native speakers. They completed high school (*n* = 67), college (*n* = 138), a two-year degree (*n* = 30), a four-year degree (*n* = 47), or a professional degree (*n* = 6) with one participant who did not complete high school and one who held a doctorate. They were enrolled in a variety of study programs and years, with first (*n* = 47), second (*n* = 66) third (*n* = 61), forth (*n* = 59), and fifth or higher (*n* = 57) year students.

**Inter-Item and Item-Total Correlations**

Inter-item and item-total correlations were small and ranged from *r* = -.07 to *r* = .32. as well as from *r* = .16 to *r* = .36, respectively. This time, the network plot (Figure 2 S3) shows a slightly clearer clustering pattern, where some risky-choice and attribute framing items are correlated with each other. However, the correlations are still small.

**Figure 2 S3**

*Network Plot of the Bivariate Correlations Between the Items of the Resistance to Framing Scale for North America (Including Outliers)*


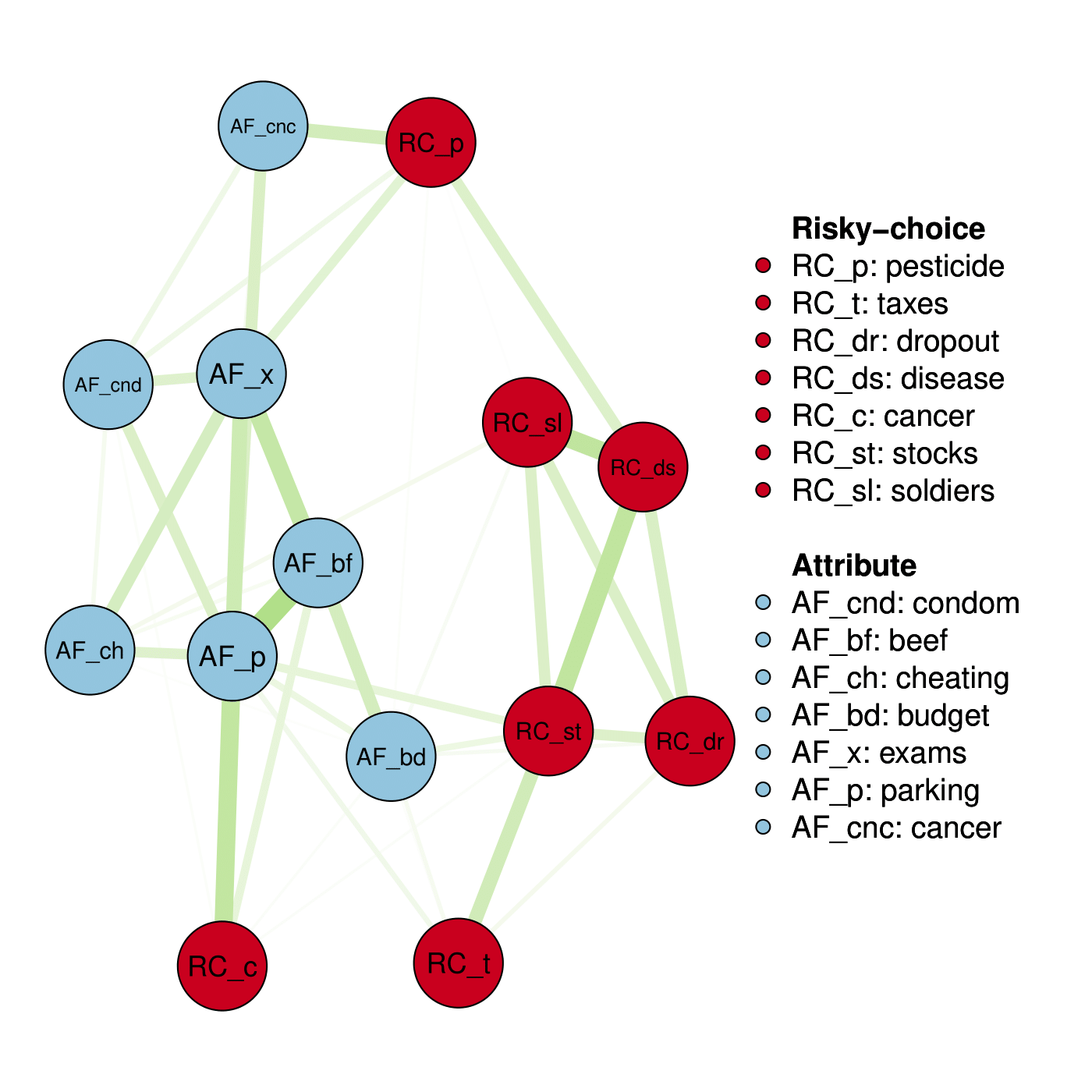


**Assumptions**

As multivariate normality was violated (*HZ* = 1.91, *p* < .001), the models were fit using robust ML estimation.

**One-Factor vs. Two-Factor Model**

The hypothesized one-factor model did not fit the data acceptably based on our preregistered criteria in Table 1. The robust χ^2^-test indicated no exact fit (χ^2^(77) = 131.63, *p* < .001, scale correction factor = 1.38). The robust RMSEA suggested acceptable fit (RMSEA = .06, 90% CI[.04, .08], SRMR = .07), whereas the robust incremental fit indices showed unacceptable fit (CFI = .67, TLI = .61, IFI = .66). However, all 14 items loaded significantly onto the RtF factor.

The hypothesized two-factor model did not fit the data acceptably. The robust χ^2^-test suggested no exact fit (χ^2^(76) = 98.05, *p* = .045, scale correction factor = 1.36). The robust RMSEA and the SRMR indicated good to excellent fit (RMSEA = .04, 90% CI[.01, .06], SRMR = .06). The robust incremental fit indices suggested questionable to poor fit (CFI = 0.87, TLI = 0.84, IFI = 0.86). Again, all 14 items loaded significantly onto their corresponding factor.

**Reliability**

Both scales showed poor reliability with a McDonald’s ω of .52 for risky-choice framing and .58 for attribute framing.

**Conclusion**

The results are partially robust to outlier exclusions. Inter-item correlations were higher–although still mostly low–for the analysis including rather than excluding outliers. Followingly, the network plot also showed a slightly clearer clustering pattern. The model fit of both models was worse for the analysis including outliers than for the one excluding outliers. For the former, all items loaded onto their corresponding factors, while for the latter, some items did not load onto the factors. The reliabilities for risk-choice framing were comparable between the analyses including and excluding outliers. The reliabilities for attribute framing were higher and almost acceptable for the analysis including rather than excluding outliers.
